# Tailored Use of Targeted Proteomics in Plant-Specific Applications

**DOI:** 10.3389/fpls.2018.01204

**Published:** 2018-08-17

**Authors:** Anja Rödiger, Sacha Baginsky

**Affiliations:** Institute of Biochemistry and Biotechnology, Martin-Luther-University Halle-Wittenberg, Halle, Germany

**Keywords:** targeted proteomics, allergen detection, breeding, food quality, food safety

## Targeted proteomics comes in different forms with the same basic principle

**Figure 1:**
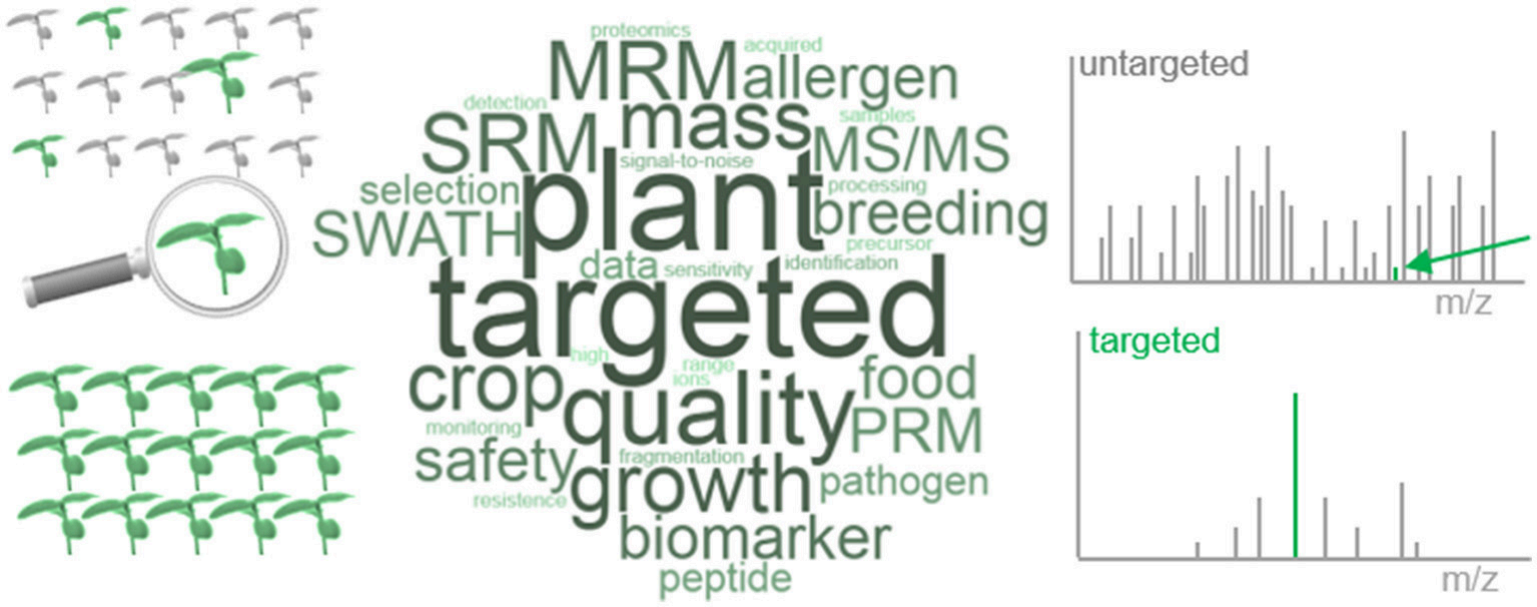
Illustrative scheme of targeted proteomics in plants. Targeted proteomics provides higher reproducibility over conventional discovery approaches resulting in higher reproducibility of analyte detection in different individuals or communities **(Left)**. This is because the dynamic range constraint of precursor selection is minimized and signal-to-noise ratios improve with the selection of a specific m/z peak for further fragmentation **(Right)**. The word cloud names some plant specific targeted proteomics applications as detailed in the main text.

Targeted proteomics comes in different flavors and the literature is full of associated technical terms such as *selected reaction monitoring* (SRM), *multiple reaction monitorin*g (MRM), *parallel reaction monitoring* (PRM), and *accurate inclusion mass screening* (AIMS) (reviewed in e.g. Picotti and Aebersold, 2012; Boersema et al., 2015; Gillet et al., 2016; Borràs and Sabidó, 2017). Recently, the data-independent acquisition method *sequential window acquisition of all theoretical fragment ion spectra mass spectrometry* (SWATH-MS) was introduced as a targeted proteomic method even though it differs considerably from the others (see below) (Gillet et al., 2012; Röst et al., 2014). The underlying rationale of all targeted proteomics approaches is the specific identification, characterization and quantification of a limited set of pre-defined peptides or proteins in an MS analysis. *Nature Methods* elected targeted proteomics method of the year in 2012 because it has great advantages over untargeted (referred to as “survey” or “discovery”) proteomics with respect to identification sensitivity and quantification reproducibility. In discovery proteomics, scans are performed over the full accessible mass range to select precursor ions for fragmentation (MS/MS). This increases the time for the identification, selection and fragmentation of precursors (duty cycle) and the dynamic range of different precursor abundances that need to be handled. Because proteomes are highly complex, precursor selection is a stochastic process making MS/MS-based peptide identifications inherently difficult to reproduce (Figure 1). In targeted approaches, the MS only scans for a set of pre-defined masses thus eliminating the stochastic nature of precursor selection and reducing duty cycle times and dynamic range constraints. Additionally, signal-to-noise ratios improve significantly, and acquisition times for peptides within a limited mass range (dwell times) can be increased to increase sensitivity (Figure 1; Borràs and Sabidó, 2017; Hart-Smith et al., 2017). Taken together targeted MS allows testing specific hypotheses with high accuracy at high reproducibility, which has led Picotti and colleagues to designate targeted proteomics as the *bridge from large-scale data acquisition to the “scientific method”* (Picotti et al., 2013a).

Targeted proteomics relies on the selection of a specific peptide, i.e. a mass over charge value in the first dimension MS. This selection is crucial for targeted MS and works by the mass filter capacities of e.g., quadrupole analysers. Targeted proteomics results improve with selection accuracy (Gallien et al., 2012, 2013). In combination with reproducible chromatography, precursor selection can be scheduled for the approximate elution time of the peptide of interest, thus increasing the multiplexing capacity of the approach. In *selected reaction monitoring* (SRM) or *multiple reaction monitoring* (MRM), a second mass filter is employed in the second dimension (MS/MS) to search for one or several specific product ions (so called “transitions”) while in *parallel reaction monitoring* (PRM), a full MS/MS spectrum is acquired from one specific m/z peak. SRM and MRM require knowledge about the best transition for every precursor and several transitions must be identified for a reliable peptide identification and quantification. Thus, cycling times between MS and MS/MS acquisitions and dwell times must be balanced to achieve good assay results. This problem is overcome in PRM where the full MS/MS spectrum is acquired and all transitions are captured in one experiment. Quantification is based on the abundance of product ions (transitions), and the gold standard for quantification includes stable isotope tagged standard peptides at known concentrations (sometimes referred to as AQUA peptides) in the analysis. The rationale of SWATH MS is significantly different from the methods described above. SWATH-MS generates high-resolution ion maps by breaking down the entire accessible mass range into smaller cascading mass windows that are sequentially scanned. Within the mass windows, all precursor ions are fragmented in a data independent manner. Consequently, all precursor ions and all fragment ions are present in the acquired ion maps. The targeted aspect of SWATH MS comes with the data analysis, i.e. specific precursor and product ion pairs of interest are extracted from the high-resolution proteome map *a posteriori*. This type of approach is feasible with all data-independent fragmentation modes including MSE (Plumb et al., 2006). The scanned mass ranges determine sensitivity and depth of such analyses, i.e., the smaller the better. Since SWATH MS aims at generating complete proteome maps, the smallest mass range is constrained by the time required to cover the entire accessible mass range by sequential scanning. Thus, unlike other targeted proteomics methods, SWATH MS misses the physical advantages that come with a narrow mass scan for precursor selection (see above).

Targeted proteomics is widespread and advanced in fields where biomarker discovery is of importance. For example, SRM assays were used to identify biomarkers for pancreatic cancer in blood plasma samples, to search for urothelial bladder cancer biomarkers in urine samples and PRM was employed for the identification of endometrial cancer biomarkers in small amounts of uterine samples (Pan et al., 2012; Martinez-Garcia et al., 2016, 2017; Duriez et al., 2017). Targeted proteomics has the sensitivity and the quantitative robustness to identify and quantify low abundance biomarkers in complex tissue samples such providing high quality data that are necessary to distinguish samples from diseased and healthy patients with high confidence. This interest in biomarkers from the medical field motivates financial investment in targeted proteomics technology and the development of tools for their application in the clinic. Plant proteomics is significantly lagging behind in the application of these tools even though there are plant-specific applications with an economic interest. Reasons lie in the small plant proteomics community, the lack of specific target peptides/proteins that would qualify as biomarkers and the *a priori* effort that is required to design targeted proteomics assays. We are nonetheless convinced that the plant field would benefit from targeted proteomics and we present here a selection of recent applications in plant-specific topics that are connected to crop plant breeding and plant growth, food quality assessment and allergen detection in processed plant material.

## Targeted proteomics in plant-specific applications: breeding, yield and food quality and safety

Breeding largely relies on DNA markers (loci) that can be associated with the establishment of a quantitative trait (QTL). Since the emergence of plant traits requires the complex interplay between different metabolic and regulatory processes and may require a specific combination of posttranscriptional, translational or posttranslational regulation, identification of protein QTLs (pQTL) is desirable to predict the outcome of genetic crosses. The feasibility to identify pQTLs with proteomics was demonstrated by different labs using e.g., crosses of genetically well-characterized mouse strains (Chick et al., 2016) or yeast (Picotti et al., 2013b). The latter study generated complete mass spectrometric maps and established an SRM assay to measure the abundances of 150 proteins over 78 *S. cerevisiae* strains. Both studies revealed complex relationships between independent genetic loci with local and distant pQTLs affecting the accumulation of proteins. In a proof-of-concept study with potato plants, Levander and colleagues showed the potential for targeted proteomics to assist in precision breeding (Chawade et al., 2016). The authors searched for peptide biomarkers in crosses from different potato varieties that allow predicting resistance against *Phytophtora infestans* while having favorable agronomic traits. They analyzed the complement of secreted proteins that show a quantitative responsiveness to exposition with this pathogen in resistant and susceptible plants by a discovery approach and defined an SRM-assay based on their quantitative data using 104 putative marker peptides. They generated synthetic peptides for quantitative reference and devised the SRM assay with the help of Skyline (Pino et al., 2017). This assay proved successful in predicting a favorable resistance vs. yield trade-off and it is now available for scale-up to characterize a larger number of different crosses.

Plants often have to cope with adverse environmental conditions necessitating rapid responses of the proteome for a suitable adaptation in cellular metabolism. The quality of the molecular response may have a dramatic impact on plant performance in the field and thus agronomic yield (Kromdijk et al., 2016). Rapid adaptations operate by modification of existing proteins, e.g., photosynthetic performance is controlled by phosphorylation of light harvesting proteins (Bennett, 1977). Research efforts with plants are therefore often devoted to unravel basic mechanisms of rapid signaling e.g., by protein phosphorylation. Phosphoproteomics in discovery mode lacks quantitative robustness unless combined with isotopic labeling or a concise analysis of quantities of isotopic clusters in label-free approaches (Schönberg et al., 2017). Targeted proteomics is capable of capturing the dynamics in this process at high resolution and with high accuracy. In this spirit, Van Ness and colleagues identified early phosphorylation events in the establishment of a symbiosis between soil-bacteria and leguminous plants. Most plants of the latter family are capable of meeting their nitrogen requirement through a symbiosis with nitrogen fixing bacteria (so called rhizobia), which has practical relevance in traditional farming where fields are rotated through various types of crops including legumes to avoid depletion of available nitrogen (nitrate). They combined a discovery-type phosphoproteomics experiment using iTRAQ for relative quantification and selected targets for SRM-assays to reach high resolution in the time domain. From a selection of forty-five phosphopeptides, Van Ness and colleagues identified five phosphopeptides that show significant changes within the first minutes of endosymbiotic signaling (Van Ness et al., 2016). Similarly, the group assessed signaling in response to dehydration (water loss) with the idea to identify genetic and chemical interventions to sustain crop yield in water-limiting environments. Their SRM data identified several regulatory proteins as specific targets for early events in dehydration responses that are now available for further exploitation (E Stecker et al., 2014).

Plants produce compounds with pharmaceutical and nutritional value, among them anthocyanins and other flavonoids that are suspected to possess health-promoting potential. Provided this potential, the latter products are determinants for the quality and market value of fruits and fruit-derived products. Song and colleagues established a targeted MRM-based assay with strawberry fruits to identify and quantify the entire set of their biosynthetic enzymes to assess their regulation during fruit ripening (Song et al., 2015). The authors correlated the quantitative protein data with transcript data, volatile production and fruit maturation and found complex regulations that provided a modified hypothesis on strawberry volatile control mechanisms. This example illustrates the potential for targeted proteomics to interrogate entire sets of biosynthetic enzymes for any natural compound pathway in plants, which is not only of commercial interest but also affects assessment of food safety by allergen detection. Food allergies are increasing worldwide and several allergenic proteins were identified and cataloged (www.allergenonline.org/) (Ahsan et al., 2016; Croote and Quake, 2016). Among them, a large proportion is derived from plant material (46%) (www.allergen.org). In order to assure food safety, reliable detection and absolute quantification methods must be applied to identify allergens in processed food material, especially because allergen levels may change with different plant growth conditions and food processing schemes. MRM was used in several instances in combination with labeled standard peptides to accurately determine allergen levels in e.g., soybean, hazelnut, wheat, and maize (Houston et al., 2011; Ansari et al., 2012; Rogniaux et al., 2015; Stevenson et al., 2015). In addition to regulatory aspects, exact quantification of allergens can aid research efforts to determine the allergenicity of foods by defining thresholds for allergic reactions.

## Conclusions

We strongly believe in targeted proteomics as the method of choice for the characterization of specific biological processes at the proteome level. It may replace the need for antibody generation (i.e., it has been termed “Mass Western” or “mass spectrometrists ELISA” by some groups) and thus offers a cost-effective alternative to traditional biochemical methods. With its scalability and quantitative reliability, more rigorous statistical testing will become possible allowing reviewers and readers to better judge the significance of an observation. With the availability of proteome maps for several organisms and improved prediction of peptide elution times, software tools such as Skyline may eliminate the need for discovery-type proteomics approaches as basis for the generation of SRM assays and allow the setup of automated workflows.

## Author contributions

All authors listed have made a substantial, direct and intellectual contribution to the work, and approved it for publication.

### Conflict of interest statement

The authors declare that the research was conducted in the absence of any commercial or financial relationships that could be construed as a potential conflict of interest.
